# Pollinator biological traits and ecological interactions mediate the impacts of mosquito-targeting malathion application

**DOI:** 10.1038/s41598-022-20823-2

**Published:** 2022-10-11

**Authors:** Dongmin Kim, Nathan D. Burkett-Cadena, Lawrence E. Reeves

**Affiliations:** Florida Medical Entomology Laboratory, University of Florida, Vero Beach, FL USA

**Keywords:** Biochemistry, Ecology

## Abstract

Mosquito adulticides are perceived by the public as detrimental to nontarget arthropods, contributing to declines of native and beneficial insects. However, the actual impact of adulticides on nontarget arthropods in nature needs to incorporate biological and ecological elements. Here, we investigated the effect of biological/behavioral traits (butterfly roosting at different heights, roosting in sites underneath foliage, bumblebee hive usage) and interactions (parasitism, predation) of pollinators (butterflies and bumblebees) that could mediate the impacts of malathion application in manipulative semi-field experiments in Florida, USA. Roosting height from the spray route had a significant negative relationship with mortality of butterflies treated with ULV malathion, with high survival at the highest roosting height (7 m), but butterflies roosting among vegetation did not have higher survival. Bumblebees with hive access had significantly higher survival than bumblebees without hive access. Host plants treated with ULV malathion significantly reduced parasitism of monarch eggs by *Trichogramma platneri,* but increased predation of monarch caterpillars by *Polistes* paper wasps. These data provide insight into the realistic impacts of adulticide applications on pollinators in nature which will enable mosquito control districts to better limit nontarget effects of adulticide treatments and may help to address concerns related to potential nontarget effects.

## Introduction

Pollinators provide critical ecosystem services through the pollination of both crops and natural plant communities^1^. Insects are particularly important pollinators, estimated to be responsible for 60–80% of all pollination worldwide^2^. Despite the recognized importance of these beneficial insects, many studies have shown that pollinators are undergoing population declines and that their ecosystem services can be substantially disrupted by human activities^3–5^.

Pollinator population dynamics are both directly and indirectly influenced by a wide range of factors within the environment including landscape properties (e.g., plant distribution, blooming phenology, habitat loss), climate change^6^ and insecticides^7,8^. Mosquito adulticides, a type of insecticide targeting adult nuisance and vector mosquitoes, have served an important role in reducing vector populations and mitigating active arbovirus transmission for public health. Since broad-spectrum adulticides (e.g., pyrethroids and organophosphates) have non-target or residual adulticide toxicity, the applications are often popularly perceived as detrimental to nontarget organisms, contributing to declines of native and beneficial insects (e.g., Springer, P. 2021. Mosquito spraying caused a ‘monarch massacre’ Can a repeat be avoided? Available from https://www.inforum.com/news/mosquito-spraying-caused-a-monarch-massacre-can-a-repeat-be-avoided [accessed 12 April 2022]) and antithetical to pollinator conservation^9^. However, research on the nontarget effects of mosquito adulticide treatments on pollinators, beyond laboratory-based direct susceptibility studies, has been limited. Beyond susceptibility to exposure, few studies quantify the direct (e.g., contact with or consumption of contaminated leaves or nectar) or indirect (e.g., predator/parasitoid-related effects) impacts of adulticides on nontarget insects within complex landscapes. A relatively large proportion of controlled studies are focused on the European honeybee, *Apis mellifera* (Linnaeus)^10^, an economically important albeit non-native pollinator and/or use artificial methodology (e.g., bottle bioassays), topically exposing insects to adulticides^11^. Such methods of exposure are likely not representative of the exposure nontarget pollinators experience under field conditions. Although the results of such assays are valuable for understanding lethal concentrations and baseline susceptibility, it is difficult to relate these data to field exposure and these laboratory-based methods do not account for factors that may be important determinants of impacts in nature such as behavioral avoidance.

Predators and parasitoids can have a strong influence over the population dynamics of their hosts^12,13^. These interactions can affect pollinator populations, particularly lepidopteran pollinators that, as larvae, are parasitized by dipteran and hymenopteran natural enemies. These parasitoids are often small, similar in size to mosquitoes, and represent another group of potential nontarget insects. It is unknown how adulticides that target mosquitoes affect these nontarget organisms, or how lepidopteran pollinators might be indirectly impacted (and potentially benefitted) by parasitoid or predator suppression, or interruptions to predator or parasitoid foraging in adulticide treated areas. Alteration of predator or parasitoid populations can have implications for prey/host species, and entire ecosystems^13,14^. Commonly, increases in prey populations follow the elimination or alteration of predator diversity and community structure, respectively. For example, a study^15^ identified more than 12 arthropod taxa (e.g., Vespidae, Chrysopidae, Formicidae) that are predators of monarch butterfly eggs and caterpillars, *Danaus plexippus* (Linnaeus), in nature. Together, these predators contributed to high mortality (40–95%) in immature monarchs^16^ suggesting that these interactions were important population drivers^17^. This top-down force extends further to the interplay between host and parasitoid interactions. Although parasitoids are considered beneficial insects within agricultural settings, in nature, their impacts can lead to population decline and even extirpation of their hosts^18^. A study^19^ showed that parasitism of monarch caterpillars by the tachinid fly *Lespesia archippivora* (Riley) was estimated to be 30% in an adulticide-free conservation area highlighting the importance of these ecological interactions on the population dynamics of the monarch.

Native pollinator communities in the USA are diverse and consist of a wide range of species of various insect orders, including several imperiled butterfly and bumblebee species. The monarch butterfly, for example, an iconic North American butterfly and focus of conservation concern, has been declining in the United States^20,21^ over the past few decades. Monarch butterflies are highly sensitive to environmental variables including drought and precipitation variability^22,23^. Degradation of habitat, particularly reduction in the abundance of their milkweed (*Asclepias* spp., and other asclepiad genera) host plants, is a primary driver of monarch population decline^4^, but insecticides in the environment can also negatively affect monarch development and survival^24,25^. Bumblebee (*Bombus* spp.) populations, another group of important pollinators, have also been significantly impacted by pesticide use across North America^26^. The use of diverse insecticides has long been debated and linked to multiple adverse sublethal effects on pollinators^27^.

Estimating the effect of mosquito adulticides on nontarget insects in nature poses many challenges due to variations in biotic (e.g., resistance, variation between species) and abiotic (e.g., temperature, precipitation) factors, but in addition to laboratory susceptibility assays, it is important to investigate the actual impacts of mosquito adulticide applications on native pollinator communities in the field to better understand and balance the risks and benefits of insecticide applications. Therefore, we explored direct and indirect impacts and various factors that may affect susceptibility of nontarget butterflies and bumblebees to understand how the natural behaviors these taxa may be affected in nature. We assessed the impacts of mosquito adulticide application (malathion) to host plants on monarch larvae, predator–prey interactions between monarch larvae and *Polistes* paper wasps, and parasitoid-host interactions between monarch eggs and *Trichogramma* egg parasitoid wasps under semi-field conditions. Also, we evaluated the effects of bumblebee nocturnal resting within a hive, butterfly roosting at different heights, and roosting underneath vegetation on mortality from malathion under field conditions. The objectives of this study were to provide information on whether behaviors of non-target pollinators (butterflies and bumblebees) affect their susceptibility to mosquito adulticide applications, and whether adulticide applications affect predator–prey and parasitoid-host interactions.

## Results

**Figure 1:**
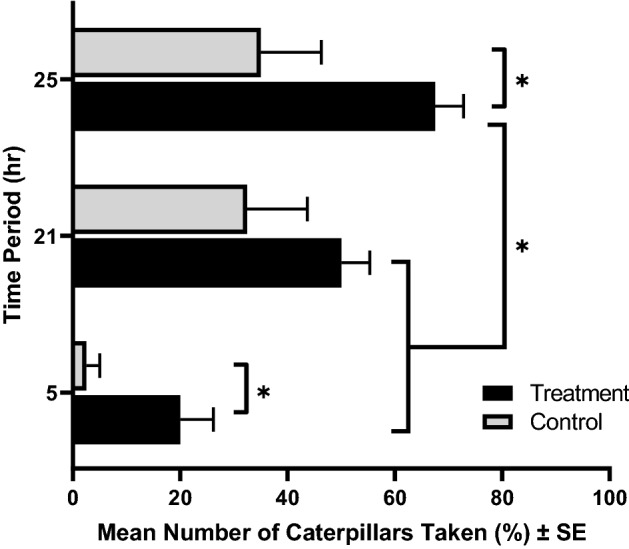
Effect of malathion on predation of monarch (*D. plexippus*) caterpillars. The rate of predation by aerial predators on monarch caterpillars (N = 4 per plant and N = 160 total) feeding on host plants treated with malathion was assessed at 5, 21 and 25 h after treatment. None of the caterpillars on host plants under mesh laundry hampers were predated. Bars indicate the mean number of caterpillars killed or taken by treatment group. Asterisks indicate significant differences between groups, using GLM with Poisson distribution (*p* < 0.05).

Predation of monarch caterpillars was greater on malathion-treated plants than on control plants (*p* < 0.001) and increased over time (Fig. 1). Paper wasp predation on monarch larvae was significantly greater for caterpillars feeding on a host plant with malathion application at 5 h (*p* = 0.012) and 25 h (*p* = 0.04) compared to the control, but the difference was not significant at 21 h (*p* < 0.001). No caterpillars on host plants under mesh hampers were predated during the entire experiment period. Mortality of malathion spray at different heights and/or distances on female *Aedes aegypti* (Linnaeus) mosquitoes is shown in Table 1.

**Table 1:** Percent mortality per cage of adult female *Ae. aegypti* mosquitoes held in cages (N = 25 per cage) at various heights and/or distances 12 h after exposure to malathion ULV spray application.

Experiment (figure #)	Height (m)	Mosquito mortality (%)			
		Spray distance (m)			
		25	50	75	Control
1	N/A	100	N/A	N/A	0
2	N/A	100	N/A	100	0
3	N/A	N/A	100	N/A	0
4	1	100	100	N/A	0
	4	100	100	N/A	0
	7	0	8	N/A	0
5	N/A	N/A	100	100	0

**Figure 2:**
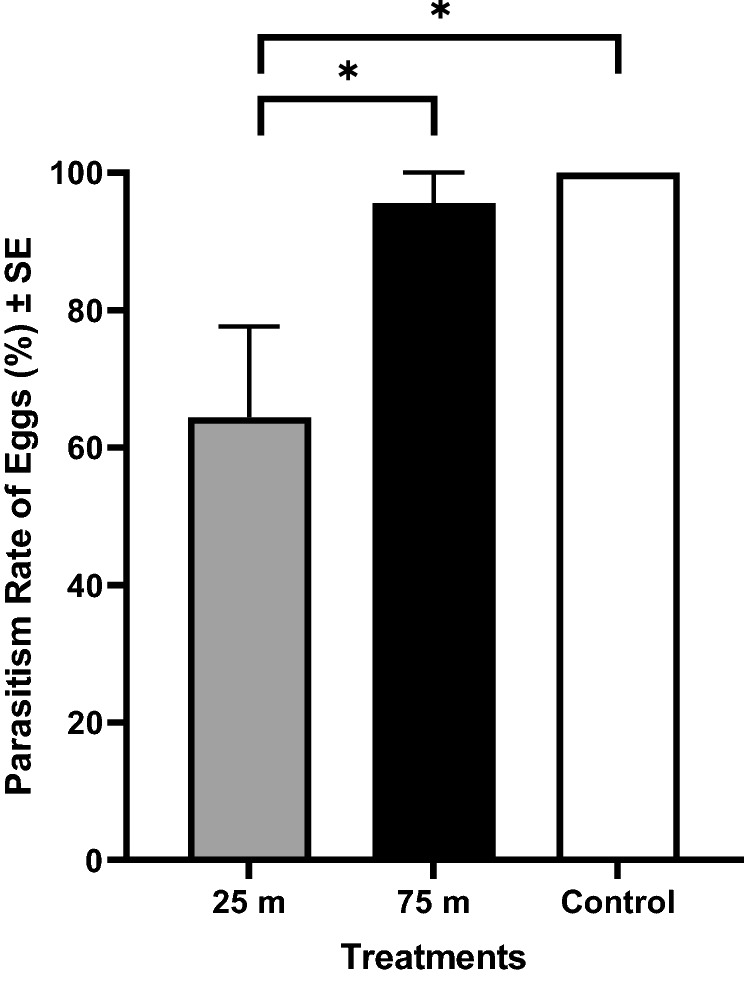
Effect of malathion on parasitism of monarch (*D. plexippus*) eggs. Rate of parasitism by *T. platneri* on monarch eggs affixed to host plant leaves (N = 5 per plant and N = 135 total) treated with malathion via truck-mounted sprayer at the maximum label rate. Asterisks indicate significant differences between groups (*p* < 0.05), using GLM with the Poisson distribution.

The rate of monarch egg parasitism by *T. platneri* was lowest when eggs were affixed to plants at the closet malathion-application distance. Parasitism rates differed significantly (*p* < 0.001) between the two treatment distances (25 and 75 m) (Fig. 2). The parasitism rate was 64.4, 95.6 and 100.0% in the 25 m, 75 m and control group, respectively. Monarch eggs attached to host plants treated with malathion 25 m from the spray path had significantly lower parasitism rates than the 75 m group (*p* < 0.001) and control group (*p* < 0.001). There was no significant difference in parasitism rates for eggs placed on 75 m and the control group. Mortality differences of *T. platneri* mortality in the 25 m, 75 m, and control group were not observed during the experiment period.

**Figure 3:**
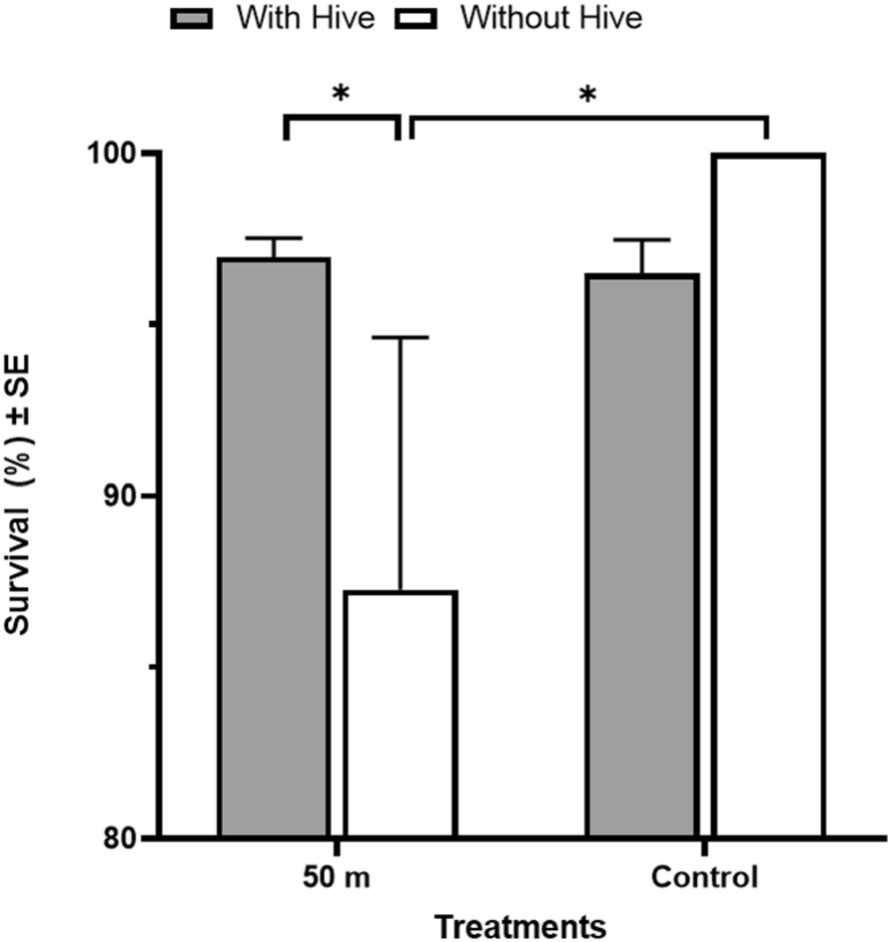
Effect of malathion on bumblebee (*Bombus impatiens*) with and without natural refugium. Survival rate of bumblebees (N = 671 total) with and without refugia (hive) at 50 m distance from truck-mounted sprayer applying malathion ULV spray at the maximum label rate. Asterisks indicate significant differences between groups (*p* < 0.05), using GLM with likelihood ratio test.

In pairwise comparisons, the survival rate of bumblebees within a hive refugium 50 m from the spray path was significantly higher than without a hive refugium (*p* < 0.001) (Fig. 3). Survival was generally high (> 85%) for all bumblebees, regardless of treatments. When access to the hive was eliminated, significantly higher number of bumblebees in the control group survived compared to those experiencing the malathion application (*p* = 0.002). Bumblebees without a hive refugium placed upwind of the spray path as control had a 14.5% higher survival rate than downwind from the spray path. There were no significant differences in survival between the malathion treated group without a hive and untreated group with a hive. When bees had access to the refugium (hive), 97–98% of bees survived regardless of treatments.

**Figure 4:**
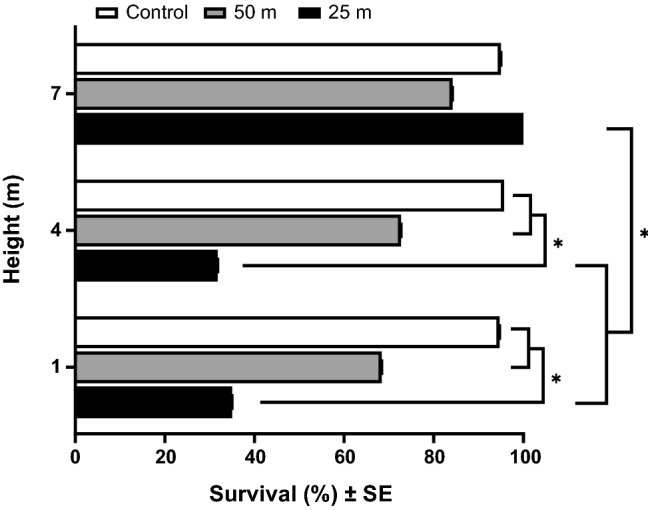
Effect of malathion on adult butterflies (N = 184 total) at different roosting heights and distances from adulticide spray route. Various species of butterfly (Nymphalidae, Pieridae and Papilionidae) were included. Asterisks indicate significant differences between groups (*p* < 0.05), using GLM with binominal distribution.

A significant interaction (*p* = 0.028) between roosting height and distance from the spray route was observed on the survival of butterflies in artificial roosts treated with malathion (Fig. 4). In general, survival decreased with decreasing height in malathion-treated groups. At 25 m from the spray path, survival between malathion ULV spray application was significantly lower for butterflies in the 1 m (*p* < 0.001) and 4 m (*p* < 0.001) roosts, compared to 7 m roosts. The highest survival (98%) was observed for butterflies roosting at the highest roosting site (7 m) at the closest spray distance (25 m), significantly higher than both 1 and 4 m heights (*p* < 0.001). Significant height and distance interaction effects (*p* = 0.027) were also observed. The survival of butterflies was not affected by the presence of a vegetation refugium (*p* = 0.664), sex (*p* = 0.529), or mass (*p* = 0.372).

**Figure 5:**
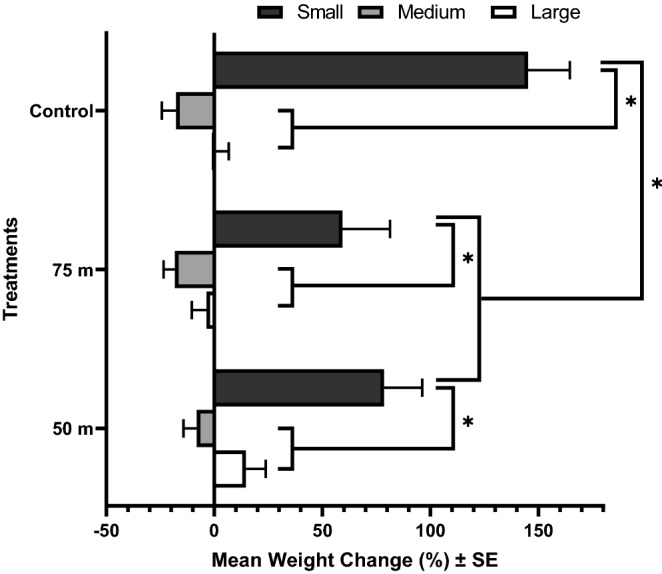
Effect of malathion on development of monarch (*D. plexippus*) caterpillars. Mean mass change of small (N = 87), medium (N = 80), or large-sized (N = 82) monarch caterpillars that fed on host plant leaves treated with malathion over 5 days. Small: < 0.07 g; Medium: 0.07–0.19 g; Large: 0.19–0.9 g. Asterisks indicate significant differences between groups (*p* < 0.05), using “indicate test”.

**Figure 6:**
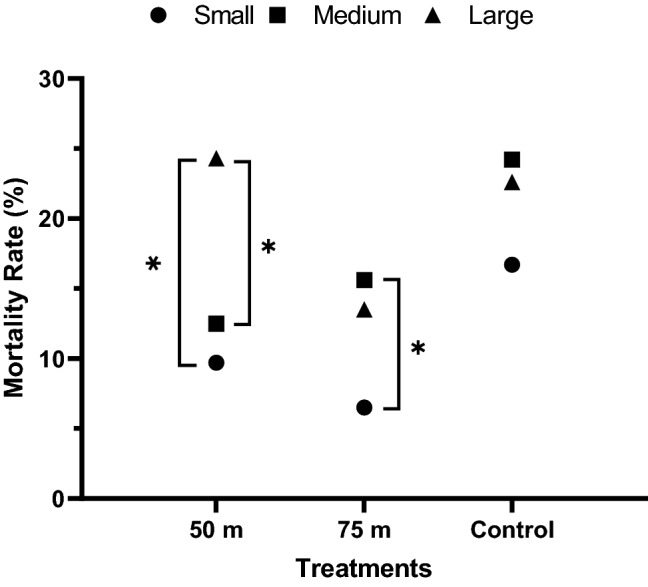
Effect of distance and size on mortality of malathion on monarch (*D. plexippus*) caterpillars. Mortality was assessed over 5 days on small (N = 87), medium (N = 80) and large (N = 82) caterpillars that fed on host plant leaves treated with malathion at 50 and 75 m distances from a truck-mounted sprayer applying a malathion ULV spray at the maximum label rate. Asterisks indicate significant differences between groups (*p* < 0.05), using GLM with the Poisson distribution.

Mass change of caterpillars was significantly affected by treatments (*p* < 0.022) and initial size (*p* < 0.001) (Fig. 5). Small caterpillars significantly increased in total mean mass across treatments (50 m, 75 m and control) over the 5 days compared to medium and large size classes. All caterpillars from the medium size class, including the control group caterpillars, decreased in mass over the 5 days. Of those from the large size class, individuals fed host plant leaves from the 50 m distance increased in mass more than others. The mortality of caterpillars from all size classes and treatments did not exceed 25% (Fig. 6). Overall, small caterpillars were resilient to feeding on host plants that had been treated with malathion ULV spray applications. Small caterpillars fed host plant leaves from the 50 and 75 m treatments experienced significantly lower mortality than large (*p* = 0.011) and medium-size (*p* = 0.047), respectively. There were no significant differences in mean weight change between small caterpillars fed host plant leaves from the 50 and 75 m distance, but the mass of small caterpillars from the control group significantly increased compared to those experiencing the malathion applications during the study period. There were no differences in mortality among size groups for untreated caterpillars.

## Discussion

We assessed factors affecting the susceptibility of non-target pollinators (butterflies and bumblebees) to mosquito adulticide (malathion) ULV applications and potential beneficial impacts to nontarget butterflies via impacts on their natural enemies. Paper wasps, *Polistes* species, specialize on lepidopteran larvae, including monarch caterpillars, which they locate, kill, and feed to their carnivorous larvae^28,29^. Because they locate caterpillars, in part, by walking over host plants^28^, we hypothesized that malathion-treated host plants would infer some protection against predation through predator avoidance of malathion-treated leaves. However, we observed a 58.6% greater predation rate for monarch caterpillars placed on malathion-treated plants compared to control groups (Fig. 1). Although we were unable to determine the reasons for this counterintuitive finding, it is possible that caterpillars feeding on adulticide-treated host plants were less able to perceive or respond to potential predators with defensive behaviors, making them more susceptible to attacks by predators. A sublethal dose of adulticide could cause paralysis or impair behavioral locomotory which may alter or discourage behavioral defensive responses to predators such as descending the host plant and moving the underside of leaves^30^, or defensive movements. Intriguingly, choice studies showed that honeybees prefer to feed on sucrose solutions containing insecticides, herbicides and fungicides^31,32^. It is also possible that caterpillars feeding on adulticide-treated host plants may be more apparent to predators. When a caterpillar has ingested adulticide, poisoning causes body effluent including regurgitation of gut contents and continual defecation which could be detected by predators as visual or olfactory cues^33^. We suggest that feeding on malathion-treated host plants does not reduce monarch caterpillar predation from these comparatively large-bodied predators. Further study is required to determine how adulticides induce modifications in behaviors (e.g., dynamics of prey/predator interaction) and physiology (e.g., detoxification mechanism).

Conversely, we found malathion application had a deterring effect on parasitism of monarch eggs by *T. platneri*, resulting in 35.6% lower parasitism for monarch eggs placed on milkweed plants treated at the closest malathion spray distance (25 m) compared to control groups (Fig. 2). *Trichogramma* species assess host quality and suitability subsequent to oviposition by walking over the leaf and egg surface to collect physical cues and chemosensory information^34^. Particularly, parasitoid species use chemical (or contact) cues or signals for host selection, oviposition and feeding and it is possible that oviposition behavior modulated by the olfactory system toward hosts (i.e., eggs) was interrupted while probing or searching hosts. It is not surprising that *Trichogramma* species may display behavioral avoidance of adulticides as these behaviors are commonly found in various other arthropod taxa including other wasps^35^, mosquitoes^36^, moths^37^ and spider mites^38^. The mechanisms responsible for behavioral avoidance may vary among parasitoid species and mode of action in adulticides^39^, but a study^40^ showed parasitism by *Trichogramma brassicae* was significantly lower in host eggs treated with deltamethrin (pyrethroids). This result suggests that a benefit may be inferred upon monarch butterflies by malathion ULV spray applications. At the same time, future work should investigate the susceptibility of monarch eggs and neonate larvae to direct exposure to malathion ULV spray treatments, and the survivorship and development of larvae feeding on such plants. A limitation of our work is that this was not assessed, and in order for this putative benefit to be likely in nature, such treatments would need to reduce parasitism with no associated adverse effects on the egg or larva. Further, the density of individual *Trichogramma* parasitoids in our experiment was artificially high, likely much higher than monarch eggs would experience in nature. For this reason, the beneficial effect we detected may be amplified in more natural settings with more realistic parasitoid densities.

Refugia protect organisms from various threats in nature including climate change, biotic (e.g., predators), and abiotic threats (e.g., drought, adulticides)^41^. We hypothesized that access to a hive as a refugium may reduce bumblebee susceptibility to mosquito adulticide-associated mortality, as adulticide spray missions take place after dark, resulting in a greater survival rate in bumblebees with hives compared to those without access to hives (Fig. 3). Bumblebees forage during daylight hours and can be exposed to adulticides through various routes including contaminated food, direct contact, or respiration^42^. However, foraging activity essentially ceases at night though sporadic, walking-based foraging may occur^43^. Although toxicity will depend on the adulticide type and concentration, our results suggest that, in nature, the nocturnal resting behaviors of bumblebees within hives reduce mortality due to mosquito adulticide applications, when these applications take place after dark, likely by reducing exposure. We were not able to provide an underground hive, as bumblebees would use in nature, however bumblebee survival within the artificial hive (provided by the supplier and intended to mimic the underground hives used in nature) was very high (> 95%). It is likely that an underground hive may provide an optimum refuge for ground-nesting bees, assuming that they are within the hive at the moment of adulticide application. The circadian activity patterns that are responsible for the daily temporal behavioral and physiological changes that can be observed in most organisms^44^ may mediate exposure and subsequent levels of mortality/survival for nontarget organisms. For example, the majority of butterfly species are day-active, resting at night. Nocturnal resting sites of butterflies vary by species, but many roost in vegetation, on leaves, terminal stems of undergrowth plants, tree trunks, or on twigs or vines underneath mats of vegetation, among others^45,46^. Some butterfly species, especially species of nymphalid subfamily Heliconiinae, roost gregariously in aggregations that provide benefits, including enhanced predator deterrence^47^ and thermoregulation^48^. Therefore, we examined the effect of nocturnal roosting behavior (i.e., roosting height and roosting amongst vegetation) on adult butterfly susceptibility to adulticide-related mortality. Although our experiment does not suggest that vegetation shields butterflies from exposure to ULV spray application, roosting height and spray distance were influential factors for survival (Fig. 4), a result supported by^49^. Adulticide efficacy is highly associated with environmental conditions which affects absorption, penetration and detoxification^50^. In particular, wind is the most important contributor to adulticide drift^51^ and potentially a significant source of exposure to nontarget insects. It is possible that height increases with increasing wind speed above the ground, which results in decreasing droplet size with less efficacy of adulticide on butterflies. Our finding that survival was greatest in the highest roost at the nearest spray distance suggests that the adulticide plume remains close to the ground for some distance immediately after application, but rises somewhat as it disperses away from the spray source.

We found monarch caterpillars to be surprisingly resilient to feeding on malathion ULV spray-treated host plants, and mortality of caterpillars from all size classes and treatments did not exceed 25% (Fig. 6). In general, the natural mortality rate of early instar monarch caterpillars is higher than in later instars^52^, but interestingly, relative mortality was lowest among the smallest caterpillars that were fed treated host plant leaves. As caterpillar size is accepted as a quantitative parameter of larval growth^25^, these caterpillars increased in mass indicating that they were able to feed and develop by feeding on malathion-treated host plants (Fig. 5). We observed relatively high natural mortality (21.2%) with a slow development time (i.e., mass) in the control group (Fig. 6), possibly caused by protozoan or viral infections (e.g., *Ophryocystis elektrosscirrha*, nuclear polyhedrosis virus)^53^ that would be expected to have a greater impact on older larvae, or caused by interactions with milkweed host plants^54^. A study^8^ showed growth rate for monarch caterpillars feeding on contaminated milkweed plants was not significantly different among adulticide (clothianidin) dose and control group. These counterintuitive results indicate factors other than dietary exposure to adulticides may have contributed to monarch caterpillar development and mortality. More data are needed to fully understand the effect of dietary malathion exposure on early-stage larval development and how these translate to changes in monarch life-history traits including pupal survival, adult emergence, and fecundity. One limitation of this experiment is that, for logistical reasons, we were unable to follow these individuals through to the adult stage. Future work should also consider whether lepidopteran larvae consuming treated host plants have similar survival to the adult stage and adult fitness compared with groups fed untreated plants.

Despite many studies focused primarily on adulticides as environmental contaminants by investigating potential negative, direct exposure-related impacts of adulticides on non-target insects, the present study suggests that in some scenarios, indirect beneficial effects to pollinators may result from adulticide applications (e.g., reduction in egg parasitism). Surprisingly, bumblebees and butterflies exposed to malathion ULV spray intended for killing adult mosquitoes were somewhat resilient, respectively, compared to mosquitoes, for which a 100% mortality rate was observed in all treated groups. Previously published studies support this conclusion. For example, field surveys have not indicated conclusively that butterfly populations in nature are adversely impacted by mosquito control adulticides. High levels of butterfly diversity have been found persisting in areas (e.g., Key West) that are frequently treated with mosquito adulticides while reduced diversity has been found in unsprayed areas (e.g., Everglades National Park). Further, higher densities of an imperiled butterfly (Bartram’s scrub hairstreak) were found in areas with active mosquito control programs that apply adulticides by ground and air^55^. Therefore, the effect of adulticides needs to be continually re-evaluated specifically considering ecological interactions and timing of applications to better delineate the actual impacts on pollinators in nature. In general, malathion resistance levels of natural enemies and pollinators is not monitored, nor reported by mosquito control or other agency, so the resistance status of the diverse insects used in this study is not known. This information would be valuable for interpreting our results, as pollinator survival was generally high (> 75%) across all experiments. Baseline information on mosquito adulticide resistance in pollinators and natural enemies would improve our understanding of the real-world nontarget impacts of adulticide applications, which will allow adulticide applicators to make more robust predictions, minimize nontarget effects of adulticide treatments, and also help to address concerns related to potential non-target effects.

## Conclusions

Mosquito adulticide applications are an essential tool for combating nuisance and vector mosquitoes. Nevertheless, mosquito control is popularly blamed for the decline of native and imperiled pollinators. Here, we assessed the influence of natural behaviors and ecological interactions of pollinators (butterflies) on their exposure and mortality to ULV adulticides. We found that roosting above 4 m and access to hives reduced ULV adulticide mortality in butterflies and bumblebees, respectively while roosting among vegetation did not. This result suggests that a benefit may be inferred upon butterflies by plant diversity, habitat structure, or age (more elevated roosting sites)^56^, which could be one of the influential factors for nontarget effects. Malathion-treated host plants provided some protection against egg parasitoids, but increased predation of monarch caterpillars by *Polistes* paper wasps. These findings suggest that pollinators may not be as negatively impacted by mosquito control practices than laboratory susceptibility assays suggest, and they reinforce the value of performing mosquito adulticide spray missions at night after diurnal pollinators are inactive. Concomitantly, the services of nocturnal pollinators are largely overlooked^57^ and future work should address how these nontarget insects may be impacted.

## Methods and Materials

Monarch butterflies were produced in a colony that was maintained at the Florida Medical Entomology Laboratory (FMEL), Vero Beach, Florida, USA from April 2020 through October 2020. Tropical milkweed (*Asclepias curassavica* L.) plants (cultivated without pesticides) were obtained from a local supplier and were used to rear monarch larvae in cages placed within a screened enclosure at FMEL where they were protected from rain and direct sunlight. The colony was established with field-collected adult female monarch butterflies caught in Vero Beach, Indian River County, Florida, USA. After establishment, adult butterflies were mated, and the colony was maintained through captive-reared adult females. To obtain eggs, field-collected or mated females were transferred to mesh cages and provided with Gatorade (Pepsico Inc., Somers, NY)-soaked cotton and milkweed bouquets (cut *A. curassavica* stems placed through the straw opening of water-filled plastic cups with lids). Eggs were collected and transferred to caterpillar rearing cages, and the newly hatched caterpillars were provided with host plants that were previously disinfected with a dilute sodium hypochlorite (NaClO, 0.074%) solution to reduce the potential of pathogen transmission via host plants. Pupae were transferred to a separate mesh cage and after eclosion adults were transferred to mesh cages provisioned with Gatorade-soaked cotton.

Malathion, an organophosphate insecticide, inhibiting acetylcholinesterase (AChE) was used for this study. To produce malathion-treated milkweed plants for subsequent experiments, potted milkweed plants were placed downwind at 25, 50 or 75 m from a predetermined spray path in an open, mowed field at the Indian River County Fairgrounds, Vero Beach, Florida. Potted milkweed host plants (5–10) were also placed upwind of the spray path to serve as a control. Malathion applications via truck-mounted ultra-low volume (ULV) spray were performed by licensed Indian River Mosquito Control District (IRMCD) personnel. Droplet size and spray flow rate were calibrated to deliver malathion at approximately 0.005–0.007 lbs/acre. Immediately following application, all milkweed plants were transported back to FMEL, with control host plants transported in a separate vehicle from treated plants to avoid any potential transfer of malathion.

Laboratory colony (> F_800_) of *Aedes aegypti* (Orlando strain, malathion-susceptible) were maintained in an environmental chamber (27.0 ± 0.5 °C, 80.0 ± 5.0% RH and 14:10 (L:D) h photoregime) at FMEL. Screen-enclosed disc cages containing 25 female *Ae. aegypti* mosquitoes were hung from a shepherd’s hook 1 m above the ground at each treatment distance (25, 50 or 75 m) as well as the control (upwind) to confirm the effectiveness of the malathion treatment on target organisms. Sugar water-soaked cotton strips were provided on mosquito disc cages and the mosquito mortality rate was recorded after 12 h.

To test the effect of malathion on predation of monarch caterpillars by *Polistes* spp. paper wasps and other aerial predators, treated and control milkweed plants from malathion applications (25 m treatment and control) were randomly assigned to one of two treatment groups. In the predator exclusion treatment, treated and control milkweed plants were covered with a mesh hamper bag that limited access to aerial predators. In the predator access treatment, treated and control milkweed plants were not covered. Potted milkweed host plants were placed in plastic trays filled with water to a depth of approximately 2.5 cm to prevent caterpillar escape. Plants were placed outdoors at the Oslo Riverfront Conservation Area and four monarch larvae (third to fifth instar) were placed on each plant (N = 160). Excised gut and head capsule on milkweed foliage were considered signs of paper wasp attack. Caterpillar numbers on each plant were counted at 5, 21 and 25 h.

To determine the effect of malathion on egg parasitism of monarchs, freshly laid monarch eggs were transferred to malathion-treated milkweed plants and exposed to egg parasitoid wasps, *Trichogramma platneri* (Nagarkatti) (Hymenoptera: Trichogrammatidae) obtained from a commercial supplier (Rincon-Vitova Insectaries Inc., Ventura, CA). Monarch eggs from the colony were excised from the potted milkweed plants upon which they were laid by cutting small square sections (< 1 cm^2^ in area) of the leaf surrounding the egg (N = 5 per plant) and gluing (Elmer’s School Glue, Elmer’s Products, Westerville, OH) the egg-leaf sections to the upper surface of a leaf from treated (25 and 75 m) or control plants. Stems holding the leaves to which monarch eggs had been affixed were inserted through the lid of a plastic cup with 75 ml water. The cups were then placed into mesh cages (60 × 60 × 60 cm; MegaView Science Education Services Co., Taichung, Taiwan) containing approximately 30,000 parasitoid wasp adults. Nine cages were given one cup/stem from each of the treatments (25 and 75 m) and control (three cups per cage, one per each treatment). The cups were removed from the cages after 24 h, and during that time, the *T. platneri* wasps had access to the monarch eggs. Egg parasitism was recorded by observing each egg under the stereomicroscope at 0, 24, 48 and 78 h.

To assess whether nocturnal resting behaviors protect bumblebees from adulticide exposure, groups of bumblebees were exposed to malathion with and without access to their hives. Bumblebees, *Bombus impatiens* (Cresson), were obtained from a commercial supplier (Koppert Biological Systems Inc., Ann Arbor, MI). Four bumblebee hives with perforations for air exchange were placed along a line perpendicular to the wind direction (downwind) at a 50 m distance from a predetermined spray path. Bumblebee hives were obtained from the supplier and were constructed from cardboard and plastic. These hives were intended to mimic the underground hives used by *Bombus* species in nature. Mesh cages holding 20 worker bees each were placed next to each hive to serve as the no-refuge treatment. Two bumblebee hives and two mesh cages were placed in a line parallel to the spray path and one of each was placed upwind to serve as a control. After truck-mounted ULV spray of malathion was carried out (as described above), all hives and mesh cages were transported back to FMEL. The mortality rate was recorded after 12 h.

To quantify the effect of roosting height on malathion-induced mortality in wild butterflies, butterfly species from multiple families (Nymphalidae: *Agraulis vanillae*, *Anartia jatrophae*, *Heliconius charithonia*, *Junonia coenia*; Pieridae: *Ascia monuste, Phoebis sennae*; Papilionidae: *Papilio glaucus, Papilio palamedes*) were captured in Marion and Indian River Counties, Florida and affixed by the wings, with wings closed, to the jute strings of each bamboo structure, at three heights (1, 4 and 7 m) with small plastic clothespins Butterflies were exposed to truck-mounted malathion treatment, as described above. Each bamboo tower consisted of a single vertical stalk of *Arundinaria gigantea* (Walter) approximately 8 m in height, with six horizontal poles, each approximately 1 m in length, extending at a right angle from the vertical stalk at 1, 4 and 7 m above ground level. Horizontal poles were attached to the vertical pole with steel angle brackets and wood screws. Jute twine extended the length of each horizontal pole as attachment points for butterflies. The bamboo structure was held upright with six guy wires of polyester rope attached to anchor points on the ground and the vertical pole between the 7 and 1 m horizontal poles. The three bamboo structures were positioned 25 and 75 m from the spray path, and one was located upwind from the spray (control). Approximately 20 butterflies (N = 184 total) including adult colony-reared monarchs and wild-collected multiple butterfly species were affixed to three bamboo towers at three heights (1, 4 and 7 m). To mimic nocturnal refugia, a sprig of saltbush, *Baccharis halimifolia* (Linnaeus), consisting of a branch with leaves was placed in front of one-quarter of the butterflies. Each butterfly was given a unique identifying number, written on the ventral surface of the wings with a permanent marker. For each, species and treatment information were recorded. One screen-enclosed disc cage containing 25 female *Ae. aegypti* mosquitoes was hung at each height on each bamboo structure alongside the butterflies to confirm the effectiveness of the malathion treatment. Once the truck-mounted ULV spray of malathion was complete, all butterflies were transferred to an assigned mesh cage with access to dilute Gatorade and transported back to FMEL. Butterfly and mosquito mortality were recorded after 12 h.

To assess the effect of mosquito adulticide on the development and mortality of monarch larvae, caterpillars were reared on malathion-treated and control milkweed plants. Prior to the malathion application, 249 caterpillars of various sizes and instars were selected from the colony, individual mass was recorded and each was placed individually into an empty paper cup covered with mesh fabric using a rubber band. Cups were assigned to three groups based on caterpillar mass: small (< 0.07 g), medium (0.07–0.19 g) and large (0.2–0.9 g) then randomly assigned to a treatment group (50 m, 75 m, or control). After milkweed plants were treated with malathion (50 m, 75 m, or control), each caterpillar was provided with milkweed leaves corresponding to their assigned treatment. Each caterpillar was checked daily, fed additional leaves from host plants of the assigned treatment group, and mortality was recorded. After 5 days, or on the day of death, the mass of each caterpillar was recorded.

We used plant materials obtained from a local supplier and cultivated without pesticide treatments (*Asclepias curassavica*). Limbs or stems of plants (*Baccharis halimifolia*, *Arundinaria gigantea*) were harvested from ornamental plants grown at the Florida Medical Entomology Laboratory (FMEL), Vero Beach, Florida, USA. No specific permits were required for the field collections of plant materials (*Asclepias curassavica*, *Baccharis halimifolia*, *Arundinaria gigantea*) and butterflies (*Agraulis vanillae*, *Anartia jatrophae*, *Heliconius charithonia*, *Junonia coenia*, *Ascia monuste*, *Phoebis sennae*, *Papilio glaucus*, *Papilio palamedes*, *Danaus plexippus*), and this study did not involve endangered or protected species. All methods were performed in accordance with the relevant guidelines and regulations.

### Statistical analysis

Effect of malathion on predation of monarch caterpillars by *Polistes* paper wasps and parasitism of monarch eggs by parasitoid *Trichogramma* wasps were analyzed by a Generalized Linear Model (GLM) with the Poisson distribution. For the effect of malathion on bumblebees with and without access to their hives at night, GLM with likelihood ratio tests (LRT) were used to assess the significance of differences between the parameter estimates. The effect of malathion on adult butterfly mortality at different roosting heights and distances was analyzed by GLM using a binomial distribution. The effect of malathion on mean mass change and mortality in monarch caterpillars was determined by factorial analysis of variance (ANOVA) and GLM with Poisson distribution, respectively. All statistical procedures were conducted by JMP Statistics, Version 15.0 (SAS Institute Inc., Cary, North Carolina, USA). Alpha was set at 0.05 for all statistical tests.

## References

[1] Garibaldi LA (2011). Stability of pollination services decreases with isolation from natural areas despite honey bee visits. Ecol. Lett..

[2] Kremen C (2007). Pollination and other ecosystem services produced by mobile organisms: A conceptual framework for the effects of land-use change. Ecol. Lett..

[3] Kluser, S. & Peduzzi, P. Global pollinator decline: A literature review. Preprint at http://archive-ouverte.unige.ch/unige 32258 (2007).

[4] Potts SG (2010). Global pollinator declines: Trends, impacts and drivers. Trends Ecol. Evol..

[5] Rhodes CJ (2018). Pollinator decline—an ecological calamity in the making?. Sci. Prog..

[6] Huang H, D'Odorico P (2020). Critical transitions in plant-pollinator systems induced by positive inbreeding-reward-pollinator feedbacks. Iscience.

[7] Krishnan N (2020). Assessing field-scale risks of foliar insecticide applications to monarch butterfly (*Danaus plexippus*) larvae. Environ. Toxicol. Chem..

[8] Bargar TA, Hladik ML, Daniels JC (2020). Uptake and toxicity of clothianidin to monarch butterflies from milkweed consumption. PeerJ.

[9] Emmel, T. C. & Tucker, J. C. In *Mosquito Control Pesticides: Ecological Impacts and Management Alternatives* (eds Emmel, T. C. & Tucker, J. C.) 105 (Scientific Publishers, 1991).

[10] Johnson RM, Ellis MD, Mullin CA, Frazier M (2010). Pesticides and honey bee toxicity–USA. Apidologie.

[11] Olaya-Arenas P, Scharf ME, Kaplan I (2020). Do pollinators prefer pesticide-free plants? An experimental test with monarchs and milkweeds. J. Appl. Ecol..

[12] Berryman AA (1996). What causes population cycles of forest Lepidoptera?. Trends Ecol. Evol..

[13] Elkinton J, Boettner G (2012). Benefits and harm caused by the introduced generalist tachinid, *Compsilura concinnata *North America. Biol. Control.

[14] Beschta RL, Ripple WJ (2016). Riparian vegetation recovery in Yellowstone: The first two decades after wolf reintroduction. Biol. Conserv..

[15] Oberhauser K, Oberhauser KS, Nail KR, Altizer SM (2015). Lacewings wasps and fliesoh my insect enemies take a bite out of monarchs. Monarchs in a Changing World: Biology and Conservation of an iconic insect.

[16] Zalucki MP, Clarke AR, Malcolm SB (2002). Ecology and behavior of first instar larval Lepidoptera. Annu. Rev. Entomol..

[17] Hermann SL, Blackledge C, Haan NL, Myers AT, Landis DA (2019). Predators of monarch butterfly eggs and neonate larvae are more diverse than previously recognised. Sci. Rep..

[18] McCoshum SM, Andreoli SL, Stenoien CM, Oberhauser KS, Baum KA (2016). Species distribution models for natural enemies of monarch butterfly (*Danaus plexippus*) larvae and pupae: Distribution patterns and implications for conservation. J. Insect Conserv..

[19] Geest EA, Wolfenbarger LL, McCarty JP (2019). Recruitment, survival and parasitism of monarch butterflies (*Danaus plexippus*) in milkweed gardens and conservation areas. J. Insect Conserv..

[20] Stenoien C (2018). Monarchs in decline: A collateral landscape-level effect of modern agriculture. Insect Sci..

[21] Crone EE, Pelton EM, Brown LM, Thomas CC, Schultz CB (2019). Why are monarch butterflies declining in the west? Understanding the importance of multiple correlated drivers. Ecol. Appl..

[22] Brower LP, Oberhauser KS, Nail KR, Altizer SM (2015). Effect of the 2010–2011 drought on the lipid content of monarchs migrating through Texas to overwintering sites in Mexico. The Monarchs in a Changing World: Biology and Conservation of an Iconic Butterfly.

[23] Thogmartin WE (2017). Monarch butterfly population decline in North America: Identifying the threatening processes. R. Soc. Open Sci..

[24] Olaya-Arenas P, Kaplan I (2019). Quantifying pesticide exposure risk for monarch caterpillars on milkweeds bordering agricultural land. Front. Ecol. Evol..

[25] Olaya-Arenas P, Hauri K, Scharf ME, Kaplan I (2020). Larval pesticide exposure impacts monarch butterfly performance. Sci. Rep..

[26] Cameron SA (2011). Patterns of widespread decline in North American bumble bees. PNAS.

[27] Epstein L (2014). Fifty years since silent spring. Annu. Rev. Phytopathol..

[28] Rayor LS, Oberhauser KS, Solensky MJ (2004). Effects of monarch larval host plant chemistry and body size on Polistes wasp predation. The Monarch Butterfly Biology and Conservation.

[29] Baker AM, Potter DA (2020). Invasive paper wasp turns urban pollinator gardens into ecological traps for monarch butterfly larvae. Sci. Rep..

[30] Castellanos I, Barbosa P (2011). Dropping from host plants in response to predators by a polyphagous caterpillar. J. Lepid. Soc..

[31] Kessler SC (2015). Bees prefer foods containing neonicotinoid pesticides. Nature.

[32] Liao L-H, Wu W-Y, Berenbaum MR (2017). Behavioral responses of honey bees (*Apis mellifera*) to natural and synthetic xenobiotics in food. Sci. Rep..

[33] Musser RO (2002). Caterpillar saliva beats plant defences. Nature.

[34] Schmidt J, Smith J (1989). Host examination walk and oviposition site selection of *Trichogramma minutum*: Studies on spherical hosts. J. Insect Behav..

[35] Ramos RS (2018). Investigation of the lethal and behavioral effects of commercial insecticides on the parasitoid wasp *Copidosoma truncatellum*. Chemosphere.

[36] Chareonviriyaphap T (1997). Pesticide avoidance behavior in *Anopheles albimanus*, a malaria vector in the Americas. J. Am. Mosq. Control Assoc..

[37] Nansen C, Baissac O, Nansen M, Powis K, Baker G (2016). Behavioral avoidance-will physiological insecticide resistance level of insect strains affect their oviposition and movement responses?. PLoS ONE.

[38] Martini X, Kincy N, Nansen C (2012). Quantitative impact assessment of spray coverage and pest behavior on contact pesticide performance. Pest Manag. Sci..

[39] Bull D, Coleman R (1985). Effects of pesticides on *Trichogramma* spp. Southwest. Entomol. Suppl..

[40] Thubru D, Firake D, Behere G (2018). Assessing risks of pesticides targeting lepidopteran pests in cruciferous ecosystems to eggs parasitoid, *Trichogramma brassicae* (Bezdenko). Saudi J. Biol. Sci..

[41] Selwood K, Zimmer H (2020). Refuges for biodiversity conservation: A review of the evidence. Biol. Conserv..

[42] Chmiel JA, Daisley BA, Pitek AP, Thompson GJ, Reid G (2020). Understanding the effects of sublethal pesticide exposure on honey bees: A role for probiotics as mediators of environmental stress. Front. Ecol. Evol..

[43] Chittka L, Williams N, Rasmussen H, Thomson J (1999). Navigation without vision: Bumblebee orientation in complete darkness. Proc. R. Soc. B.

[44] Young MW, Kay SA (2001). Time zones: A comparative genetics of circadian clocks. Nat. Rev. Genet..

[45] Mallet J (1986). Gregarious roosting and home range in Heliconius butterflies. Natl. Geogr. Res..

[46] Chang Y-M (2020). Roosting site usage, gregarious roosting and behavioral interactions during roost-assembly of two Lycaenidae butterflies. Zool. Stud..

[47] Vulinec K, Evans DL, Schmidt JO (1990). Collective security aggregation by insects as a defence. Insect Defences. Adaptive Mechanisms of Prey and Predators.

[48] Salcedo C (2010). Environmental elements involved in communal roosting in Heliconius butterflies (Lepidoptera: Nymphalidae). Environ. Entomol..

[49] Giordano BV, McGregor BL, Runkel AE, Burkett-Cadena ND (2020). Distance diminishes the effect of deltamethrin exposure on the monarch butterfly, *Danaus plexippus*. J. Am. Mosq. Control Assoc..

[50] Matzrafi M (2019). Climate change exacerbates pest damage through reduced pesticide efficacy. Pest Manag. Sci..

[51] Hewitt A (2000). Spray drift: Impact of requirements to protect the environment. Crop Prot..

[52] Nail KR, Stenoien C, Oberhauser KS (2015). Immature monarch survival: Effects of site characteristics, density and time. Ann. Entomol. Soc..

[53] Payne CC, Mertens PP, Joklik K (1983). Cytoplasmic polyhedrosis viruses. The Reoviridae.

[54] Zalucki MP (2001). It’s the first bites that count: Survival of first-instar monarchs on milkweeds. Austral. Ecol..

[55] Salvato M (2001). Influence of mosquito control chemicals on butterflies (Nymphalidae, Lycaenidae, Hesperiidae) of the lower Florida keys. J. Lepid. Soc..

[56] Frey DF, Leong KL (1993). Can microhabitat selection or differences in ‘catchability’ explain male-biased sex ratios in overwintering populations of monarch butterflies?. Anim. Behav..

[57] Macgregor CJ, Scott-Brown AS (2020). Nocturnal pollination: An overlooked ecosystem service vulnerable to environmental change. Emerg. Top. Life Sci..

